# Encapsulating fibrosis following neoadjuvant chemotherapy is correlated with outcomes in patients with pancreatic cancer

**DOI:** 10.1371/journal.pone.0222155

**Published:** 2019-09-06

**Authors:** Yoko Matsuda, Yosuke Inoue, Makiko Hiratsuka, Shoji Kawakatsu, Tomio Arai, Kiyoshi Matsueda, Akio Saiura, Yutaka Takazawa

**Affiliations:** 1 Oncology Pathology, Department of Pathology and Host-Defense, Faculty of Medicine, Kagawa University, Kagawa, Japan; 2 Department of Pathology, Tokyo Metropolitan Geriatric Hospital, Tokyo, Japan; 3 Department of Digestive and HBP Surgery, Cancer Institute Hospital, Japanese Foundation for Cancer Research, Tokyo, Japan; 4 Department of Radiology, Cancer Institute Hospital, Japanese Foundation for Cancer Research, Tokyo, Japan; 5 Department of Pathology, Cancer Institute Hospital, Japanese Foundation for Cancer Research, Tokyo, Japan; Centro Nacional de Investigaciones Oncologicas, SPAIN

## Abstract

Pathological assessments of the treatment effect are critical for predicting patient outcomes after surgery. This study included 82 localized pancreatic cancer, 40 of whom were treated with neoadjuvant therapy (NAT) using four courses of gemcitabine plus nab-paclitaxel (GnP) followed by pancreatectomy (GnP group). The remaining 42 patients were treated with upfront pancreatectomy (UP) followed by adjuvant chemotherapy (UP group). We reviewed clinicopathological data of these patients to assess differences between the GnP and UP groups and further evaluate the prognostic impact of residual tumors after GnP treatment. Adjuvant treatment (S1, GnP or gemcitabine) was administered for 36 patients in the GnP group and 33 patients in the UP group. Compared to the UP group, the GnP group showed lower serum CA19-9 levels, microscopic tumor volume, and tumor-stroma ratio and decreased number of lymph node metastasis and vascular invasion. Higher incidence of encapsulating fibrosis was observed in the GnP group than in the UP group. Relative to the UP group (69%), a higher R0 rate was observed in the GnP group (85%). As for prognosis, encapsulating fibrosis was correlated with the overall survival of patients in the GnP group. However, overall survival did not show any correlation with other clinicopathological factors, including tumor reduction ratio (determined by computed tomography) and tumor regression grade (determined following criteria of Evans’ grading system or those of the College of American Pathologists). In conclusion, the present study revealed that GnP-induced encapsulating fibrosis could predict patients’ outcome. Nevertheless, large cohort studies are warranted to further evaluate the prognostic value of fibrosis, possibly with the help of imaging and biomarkers.

## Introduction

Owing to aggressive local growth and distant metastasis, only 15%-20% of pancreatic cancer patients can be treated surgically. Even in patients with a completely resected tumor, there is a high frequency of recurrence and low 5-year survival rate of 20%.[1] Gemcitabine has been considered the standard therapeutic agent used in both adjuvant therapy and treatment of metastatic disease [2, 3]; however, recently FOLFIRINOX [4] or a combination of gemcitabine and nab-paclitaxel (GnP)[5] showed a substantial improvement in survival compared to gemcitabine monotherapy. Neoadjuvant therapy (NAT) has been shown to improve the outcome of pancreatic cancer treatment [6–10] by increasing the proportion of patients that can be treated surgically to reduce the tumor size. Additionally, NAT might eradicate micrometastasis in remote organs.[11]

Pathological analysis of tumor tissue can facilitate the assessment of viability and changes in cancer cells following NAT; this would enable us to examine the effects of treatment in various carcinomas, including those of the esophagus, stomach and colorectum.[12] There have also been several reports studying the effects of NAT in pancreatic cancer patients.[8–10, 13] Pathological complete response (with no remnant cancer cells) to NAT in pancreatic cancer has been associated with improved prognosis.[10] Chatterjee et al. reported that during post-therapy, an incidence of perineural and intraneural invasion or muscular vessel invasion predicts poor prognosis of pancreatic cancer.[8, 9] Post-therapy pathological stage and number of positive lymph nodes are independent prognostic factors for survival in pancreatic cancer.[13]

There are several grading systems often used to assess tumor regression; however, these methodologies are known to yield inconsistent results. The grading system put forth by Evans [14, 15] and the Japan Pancreas Society (JPS) are commonly used in Japan,[16] and both grading systems focus on the degree of cellular necrosis. The College of American Pathologist (CAP)[15] guidelines are commonly followed in the United States, and they focus on the proportion of remnant cancer cells. According to Evans’ criteria, patients with grade 2b or higher (containing >51% dead cells) have a better prognosis.[17]

Chun et al. used a different method to evaluate the fibrotic areas in pancreatic cancer tissues of patients undergoing neoadjuvant therapy, and they found that a high level of fibrosis correlates with better prognosis.[18] GnP treatment has been previously reported to reduce the number of cancer-associated fibroblasts and cause alterations in cancer stroma.[19, 20] Fibrosis, inflammation, and necrosis are common characteristics of cancers resulting as a consequence of the high proliferation of cancer cells and stromal cell reaction. Therefore, it is difficult to distinguish treatment-related pathological changes from tumor proliferation-related changes, especially in pancreatic cancer.

Tumor regression can also be estimated by serial observation of the affected organs using endoscopy or radiological imaging. However, imaging examinations are not helpful in determining tumor regression in pancreatic cancers because fibrosis is a common feature shared by cancer and chronic pancreatitis. Furthermore, there are limited numbers of clinically available markers to evaluate the treatment effect in pancreatic cancers.[21, 22]

In this study, we compared the pathological changes occurring in pancreatic cancer cells as well as stroma in patients receiving NAT and those treated with upfront surgery; this enabled us to identify NAT-related pathological changes in pancreatic cancers. Furthermore, the correlation between pathological characteristics and overall survival was analyzed to understand the prognostic impact of NAT.

## Materials and methods

### Patients and tissues

In this study, we used tissues from patients with pancreatic cancer who had undergone surgery at the Cancer Institute Hospital of the Japanese Foundation for Cancer Research between the years 2015 and 2017. This study included 82 patients (Table 1); of these, 40 patients were treated with NAT using four courses of GnP followed by pancreatectomy (GnP group). Surgery was performed approximately one month after the last session of neoadjuvant chemotherapy. For the control group, we recruited 42 patients with pancreatic cancer who had not received any treatment prior to surgery (upfront pancreatectomy), followed by administration of adjuvant chemotherapy (UP group). We reviewed the clinical data [including serum tumor marker levels (carcinoembryonic antigen, CEA and carbohydrate antigen 19–9, CA19-9) and CT tumor volume) of these patients]; further, the differences were assessed for the GnP and UP groups, and the prognostic impact of residual tumors was evaluated following GnP treatment. Pre-operative resectability (resectable, borderline resectable, and unresectable) was determined by more than two radiologists, using CT or magnetic resonance imaging according to the National Comprehensive Cancer Network (NCCN) Guidelines 2014.[23]

**Table 1:** Clinical characteristics of patients treated with neoadjuvant therapy and upfront surgery.

	GnP	UP	
	Median (min-max)	Median (min-max)	P value
Age (years)	66.5 (36–81)	69.5 (48–85)	0.0582 ^a^
Pre-treatment CEA level (ng/mL)	3.2 (0.8–23.3)	2.9 (0.5–16.6)	0.2377 ^a^
Post-NAT CEA level (ng/mL)	3.0 (0.8–13.1)		0.4636 ^b^
Pre-treatment CA19-9 level (U/mL)	279.1 (2.0–8688.4)	95.1 (2.0–16375.0)	0.0159 ^a^ *
Post-NAT CA19-9 (U/mL)	23.8 (2.0–993.0)		0.0067 ^b^ *
Pre-treatment tumor volume (CT, mm^3^)	30929 (7200–34764)	22643 (630–80784)	0.0436 ^a^ *
Post-NAT tumor volume (CT, mm^3^)	18320 (1170–226920)		0.2332 ^b^
	Number (%)	Number (%)	
Sex			
Male	15 (38)	28 (67)	0.0082 *
Female	25 (63)	14 (33)	
Resectability			
R	0 (0)	31 (74)	<0.0001 *
BR	38 (95)	11 (26)	
UR	2 (5)	0 (0)	
Operation			
PD-PVR	26 (65)	38 (90)	0.0006 *
PD	1 (3)	3 (7)	
DP-CAR	13 (33)	0 (0)	
DP	0 (0)	1 (2)	
Adjuvant	36 (90)	33 (79)	0.230
Reason for absence of adjuvant treatment			
Palliative therapy for recurrence	2^c^	2^c^	
Poor postoperative recovery	2	4	
Sequential treatment for other disease	0	1	
Patient’s will	0	2	
Regimen for adjuvant			
S1	29 (73)	31 (74)	
GnP	6 (15)	1 (2)	0.1725
Gem	1 (3)	1 (2)	

*P<0.05 by Mann-Whitney U test or chi-square tests.

^a^, Comparison between pre-NAT in GnP and preoperative in UP;

^b^, Comparison between post-NAT in GnP and preoperative in UP.

^c^, Two patients were received chemotherapy as a palliative therapy. GnP, gemcitabine plus nab-paclitaxel; UP, upfront surgery; NAT, neoadjuvant treatment; CEA, carcinoembryonic antigen; CA19-9, carbohydrate antigen 19–9; CT, computed tomography; R, resectable; BR, borderline resectable; UR, unresectable; PD, pancreatoduodenectomy; PVR, portal vein resection; DP, distal pancreatectomy; CAR, celiac artery resection; Gem, gemcitabine.

Pancreatectomy specimens were cut into 5 mm slices, and all pancreatic samples were prepared as formalin-fixed paraffin-embedded specimens. Tissue samples were stained using hematoxylin and eosin (H&E) staining solution. Elastica van Gieson staining was performed to detect the presence of vascular invasion.

Herein, we conducted a retrospective study using pathological tissues from surgically resected specimens. Informed written consent to use the tissues was obtained from all patients. This study was approved by the ethics committee of the Cancer Institute Hospital, Japanese Foundation for Cancer Research (permit #2015–1084).

### Chemotherapy

For neoadjuvant therapy, four courses of GnP (intravenous injection of gemcitabine at a dose of 1000 mg/m^2^ and nab-paclitaxel at a dose of 125 mg/m^2^ over 30 min on days 1, 8, and 15, followed by a 1-week rest period) were used as a standard regimen.

Adjuvant chemotherapy was administered to both groups after we confirmed that the patient’s recovery was enough to undergo outpatient chemotherapy based on oral intake, performance status, and absence of intractable diarrhea. S-1 (oral dose of 40 mg for a body-surface area less than 1·25 m^2^, 50 mg for a body-surface area of 1·25 m^2^ or more but less than 1·5 m^2^, or 60 mg for a body-surface area of 1·5 m^2^ or more, twice per day for 28 consecutive days followed by a 14-day rest) was used as a standard regimen with some modification according to the patient’s state. The duration of adjuvant chemotherapy was usually set as 6 months. For patients with early recurrence, GnP was used as the first choice for palliative therapy.

### Pathological evaluation

More than two pathologists evaluated the parameters used for processing pancreatic specimens, according to the 7th edition of the General Rules for the Study of Pancreatic Cancer by the JPS. Carcinoma <1 mm from any resection margin was considered to be incompletely excised and was defined as R1 according to the Royal College of Pathologists.[24] With the help of microscopic imaging cancer cells were mapped and tumor diameter was determined. Using microscopic measurements, we calculated the tumor volume using the following equation: major axis × minor axis × length.

**Fig 1:**
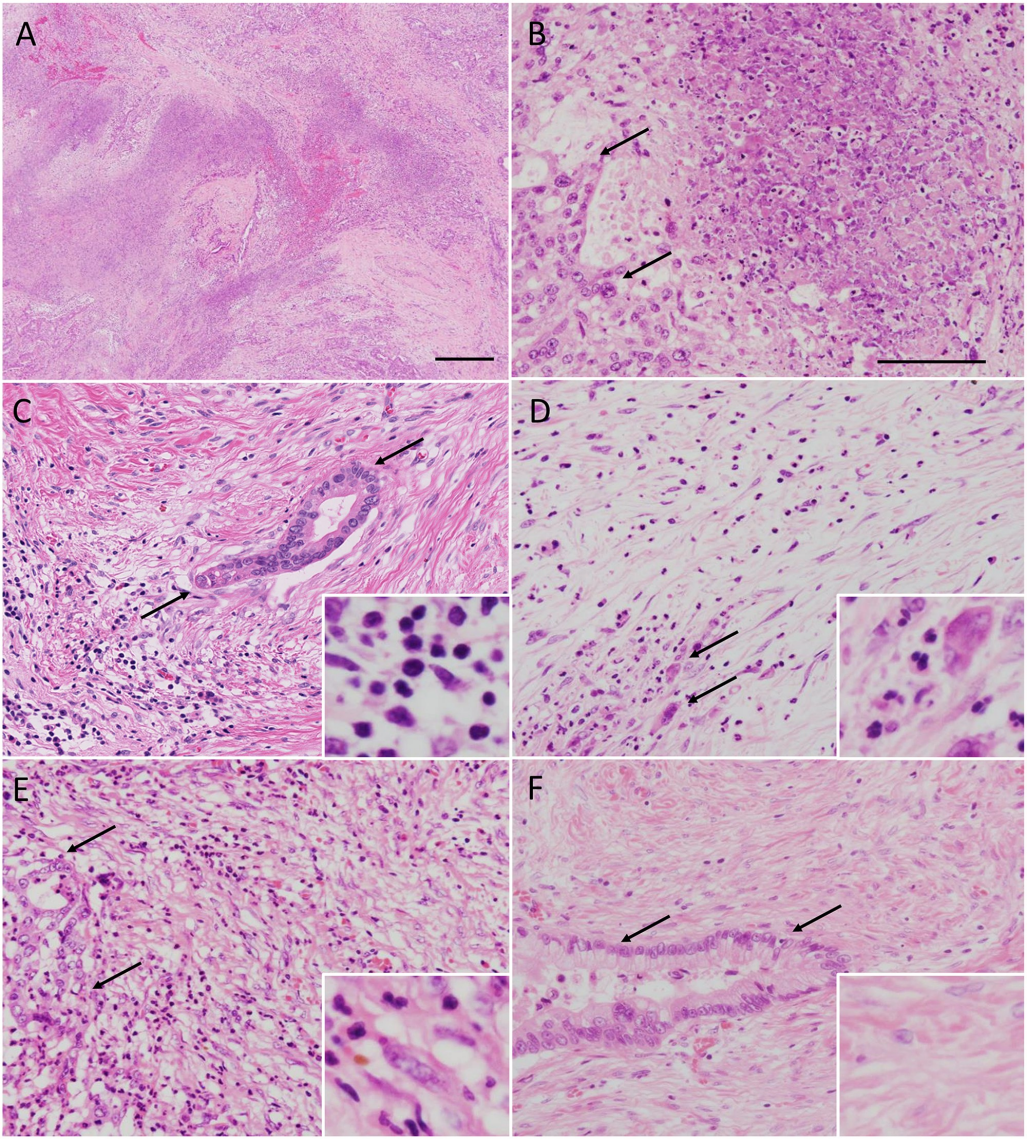
Histologic changes in pancreatic cancer patients following neoadjuvant therapy. (A) Appearance of necrosis in the areas affected by cancer. (B) Higher magnification of a region from panel (A). Arrows mark viable cancer cells. Images showing lymphoplasmacytic and neutrophilic infiltration of the stroma (C and D, respectively). Examples of immature and mature fibrosis are shown in panels (E) and (F), respectively. Arrows indicate cancer cells. All tissue specimens were stained with hematoxylin and eosin staining solution. Bars, A, 500 μm; B-F, 100 μm.

**Fig 2:**
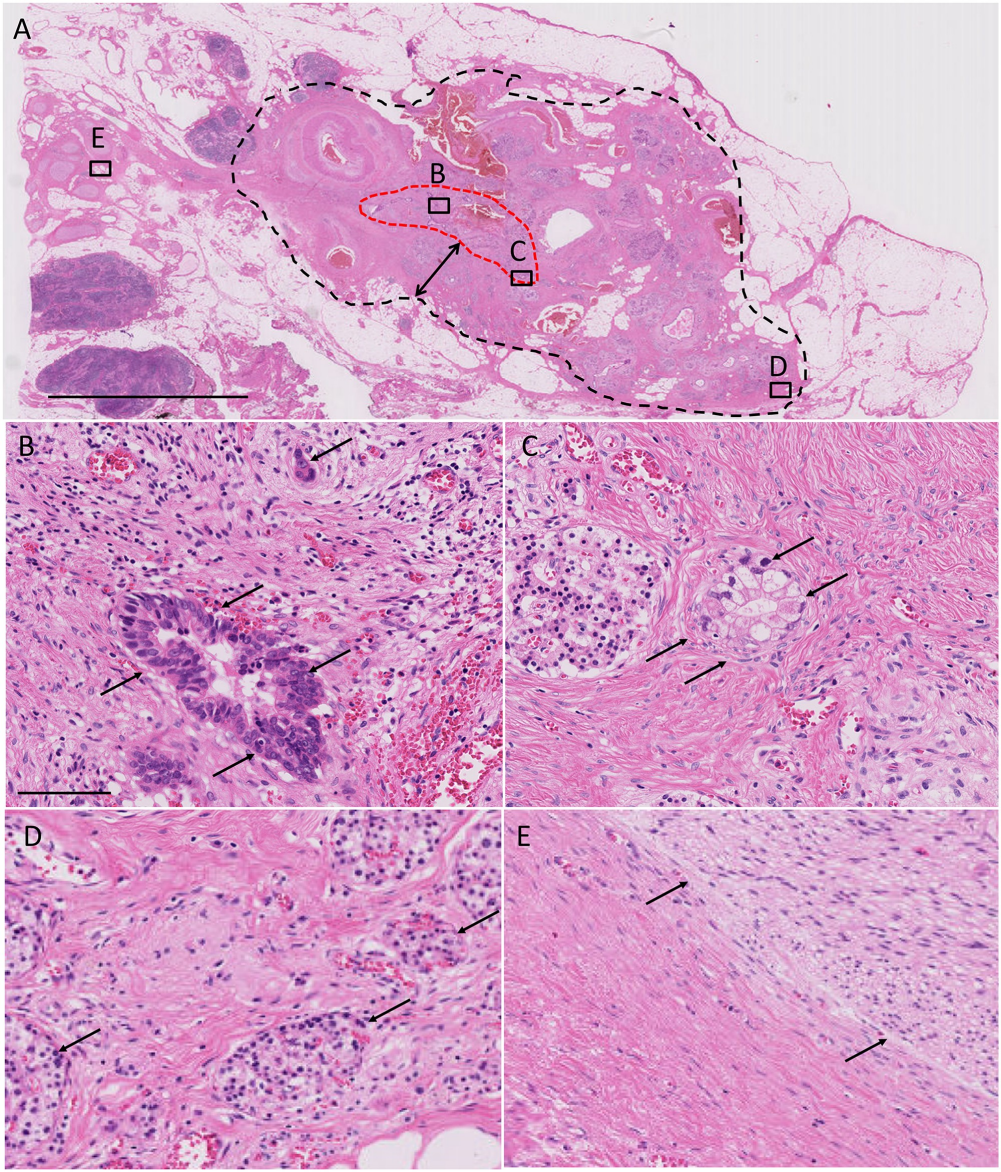
Encapsulating fibrosis following neoadjuvant therapy. (A) Red and black dotted lines represent cancer remnants and encapsulating fibrosis area, respectively. Double-headed arrow marks a fibrous area of 2 mm thickness. Panels (B-E) show the higher magnification images of the selected areas (black squares) in (A). Arrows show cancer cells (B and C), islets (D), and nerve plexus in panels (E), respectively. All specimens were stained with hematoxylin and eosin solution. Bars, A, 6 mm; B-E, 100 μm.

Following Evans’ and JPS grading systems and using the largest tumor specimen, we determined the percentage of necrotic tumor cells in the tumor area (Fig 1A and 1B). To evaluate treatment-related pathological features, we used the largest tumor specimen to score for the following histologic findings in the cancerous areas: tumor-stroma ratio, inflammation, and fibrosis. To study the inflammatory responses, we performed an analysis of the predominant tumor-infiltrating cells and demonstrated the presence of lymphoplasmacytic (Fig 1C) and neutrophilic infiltration (Fig 1D). Based on the existence of collagen, myxoid stroma, and fibroblasts, we classified fibrosis into two categories (immature, Fig 1E and mature, Fig 1F).[25] Immature fibrosis was defined as an amorphous stromal substance composed of an amphophilic or slightly basophilic material usually intermixed with randomly oriented collagen fibers (Fig 1E). Mature fibrosis was defined as fine collagen fibers stratified into multilayers with few or no fibroblasts (Fig 1F), with an occasional appearance of eosinophilic hyalinization. In addition, we evaluated the existence of encapsulating fibrosis under 10× magnification (Fig 2A, black dotted line). We defined encapsulating fibrosis by the formation of >2 mm (diameter of the field, under 10× magnification) fibrous bands surrounding cancerous area.

Following NAT, tumor regression grade was assigned according to the criteria of the JPS, Evans, and CAP. Pathological specimens were reviewed by three authors (Y.M., M.H., and S.K.) who were blinded to the clinical and patient outcome data.

### Statistical analysis

Data are presented as the median (range). The Mann-Whitney U test was used to determine the statistically significant differences between the mean values. Chi-square and Fisher’s exact tests were used to analyze clinicopathological features. Kaplan-Meier analysis was performed to analyze the relationship to overall survival and clinicopathological features. Overall survival was defined as period from surgery to death or censor. Statistical analysis was performed using the Stat View J version 5.0 software package (SAS Institute, Inc., Cary, NC, USA). A P value of <0.05 was considered statistically significant.

## Results

### Clinical characteristics of GnP and UP patients

Since the selection of our treatment strategy methods was based on resectability, there was a selection bias that resulted in the GnP group mainly consisting of patients with borderline resectable cancers (95%) while the UP group mainly comprised patients with resectable cancer (74%; Table 1). Compared with patients receiving UP, those receiving GnP treatment were predominantly female (P = 0.0082) and had higher serum CA19-9 levels (P = 0.0159, pre-NAT) and larger tumor volume (P = 0.0436). Relative to the pre-treatment levels of CA19-9, post-NAT CA19-9 levels were significantly lower in the GnP group (P = 0.0067). The initial significant differences in tumor volume determined by CT disappeared after neoadjuvant treatment (P = 0.2332).

Sixty-nine patients (84%) underwent adjuvant therapy with a median interval of 68 days after surgery (range, 40–151 days). The median duration of adjuvant chemotherapy was 6 months (1–12 months). Induction rate of adjuvant therapy was 90% in the GnP group and 79% in the UP group, respectively (P = 0.23). Among 13 patients without adjuvant therapy, 4 underwent palliative chemotherapy for early recurrence, 6 were judged intolerant of adjuvant therapy due to poor postoperative recovery, 1 underwent sequential treatment for myelodysplastic syndrome, and 2 decided to omit adjuvant therapy by their own will.

### Pathological characteristics of GnP and UP patient groups

**Fig 3:**
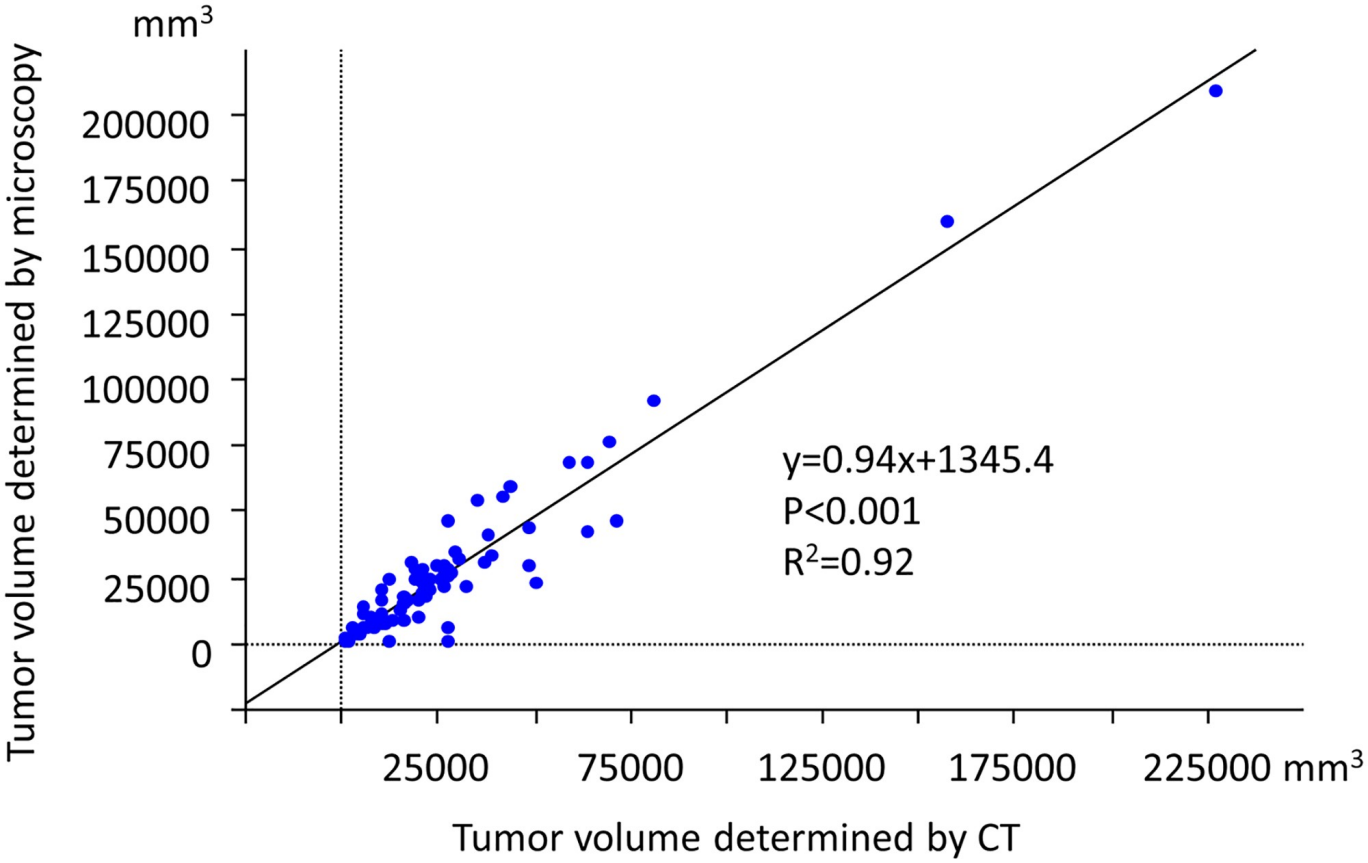
Correlation between tumor volumes determined by microscopy and CT (post neoadjuvant therapy). P<0.0001. R^2^ = 0.92.

Microscopic examinations revealed that tumor volume was smaller in GnP versus UP patients (P = 0.0471; Table 2), and microscopic tumor volume was strongly correlated with tumor volume determined by CT (post-NAT, P<0.0001, R^2^ = 0.92; Fig 3). The number of lymph nodes with metastasis was lower in patients from the GnP relative to UP group (P = 0.0341). Both the GnP and UP groups had comparable percentage of necrotic cancer cells (7.5% and 5.0% in GnP and UP groups, respectively; Fig 1A and 1B). Patients in the GnP group showed a lower tumor-stroma ratio than those in the UP group (P = 0.0053). Compared with the UP group, the GnP group had a lower incidence of vascular invasion in the pancreas (P = 0.0418). GnP patients appeared to show a scirrhous pattern that was not seen in UP patients (P = 0.0746). Additionally, the GnP group displayed a low incidence of lymphatic invasion (P = 0.0533) and positive surgical margin (P = 0.0870; 85% and 69%, respectively).

**Table 2:** Histologic difference between patients treated with neoadjuvant therapy and upfront surgery.

	GnP	UP	
	Median (min-max)	Median (min-max)	P value
Microscopic tumor volume (mm^3^)	16950 (825–209300)	24240 (770–92500)	0.0471 *
Lymph nodes with metastasis (n)	1 (0–6)	2 (0–20)	0.0341 *
Necrosis (%)	7.5 (5–70)	5.0 (0–60)	0.8845
Tumor-stroma ratio	40 (5–90)	55 (30–90)	0.0053 *
	Number (%)	Number (%)	
Histologic differentiation			
Well	11 (28)	15 (36)	0.4112
Moderate	23 (58)	18 (43)	
Poor	6 (15)	9 (21)	
Stromal pattern			
Intermediate	31 (78)	31 (74)	
Medullary	4 (10)	10 (24)	0.0746
Scirrhous	5 (13)	1 (2)	
Lymphatic invasion			
0	5 (13)	3 (7)	0.0533
1	31 (78)	25 (60)	
2	4 (10)	10 (24)	
3	0 (0)	4 (10)	
Vascular invasion			
0	1 (3)	2 (5)	0.0418 *
1	27 (68)	17 (40)	
2	7 (18)	19 (45)	
3	5 (13)	4 (10)	
Surgical margin			
0	34 (85)	29 (69)	0.0870
1	6 (15)	13 (31)	
T			
1	0 (0)	2 (5)	0.3495
2	0 (0)	1 (2)	
3	38 (95)	38 (90)	
4	2 (5)	1 (2)	
N			
0	15 (38)	10 (24)	0.1783
1	25 (63)	32 (76)	
Inflammation			
Lymphoplasmacytic	9 (23)	10 (24)	0.8883
Neutrophilic	31 (78)	32 (76)	
Fibrosis in the tumor			
Mature	34 (85)	27 (64)	0.0317 *
Immature	6 (15)	15 (36)	
Encapsulating fibrosis			
Negative	14 (35)	37 (88)	<0.0001 *
Positive	26 (65)	5 (12)	

*, P<0.05 between GnP and UP groups by Mann-Whitney U test or chi-square tests. Pathological scores were determined according to General Rules of Japan Pancreas Society, 7th edition. T and N stages were determined according to UICC 8th edition. GnP, gemcitabine plus nab-paclitaxel; NAT, neoadjuvant treatment; UP, upfront surgery.

The inflammation patterns (i.e., predominantly lymphoplasmacytic, Fig 1C or neutrophilic, Fig 1D) were not significantly different between the two groups. However, the fibrosis patterns, i.e., immature (Fig 1E) or mature (Fig 1F) fibrosis, were significantly different between the two groups; relative to the UP group, GnP group had a higher incidence of mature fibrosis in cancerous areas (P = 0.0317, Table 2). We found that the GnP patient group often developed mature fibrosis in the area with remnant cancer (Fig 2A; red dotted line marks the area affected by cancer). In these patients, both patterns of fibrosis (immature fibrosis, Fig 2B and mature fibrosis, Fig 2C) were observed in the cancerous area; furthermore, they had extended to the pancreatic parenchyma (Fig 2D), as well as in the peripancreatic fat tissue and nerve plexus (Fig 2E). We observed mature fibrosis without cancer cells in one microscopic field under 10× magnification (2 mm diameter; Fig 2A, marked by double-headed arrows); therefore, the development of such lesions was described as "encapsulating fibrosis". The presence of encapsulating fibrosis was 65% and 12% in the GnP and UP groups, respectively (P<0.0001, Table 2).

In this study, we observed the presence of mucous pools in three GnP cases, macrophage aggregation in one GnP and one UP case, calcification in two GnP cases, and the clear-cell variant of cancer in four GnP and two UP cases.

### The relationship between clinical and conventional pathological characteristics and overall survival of GnP patients

**Fig 4:**
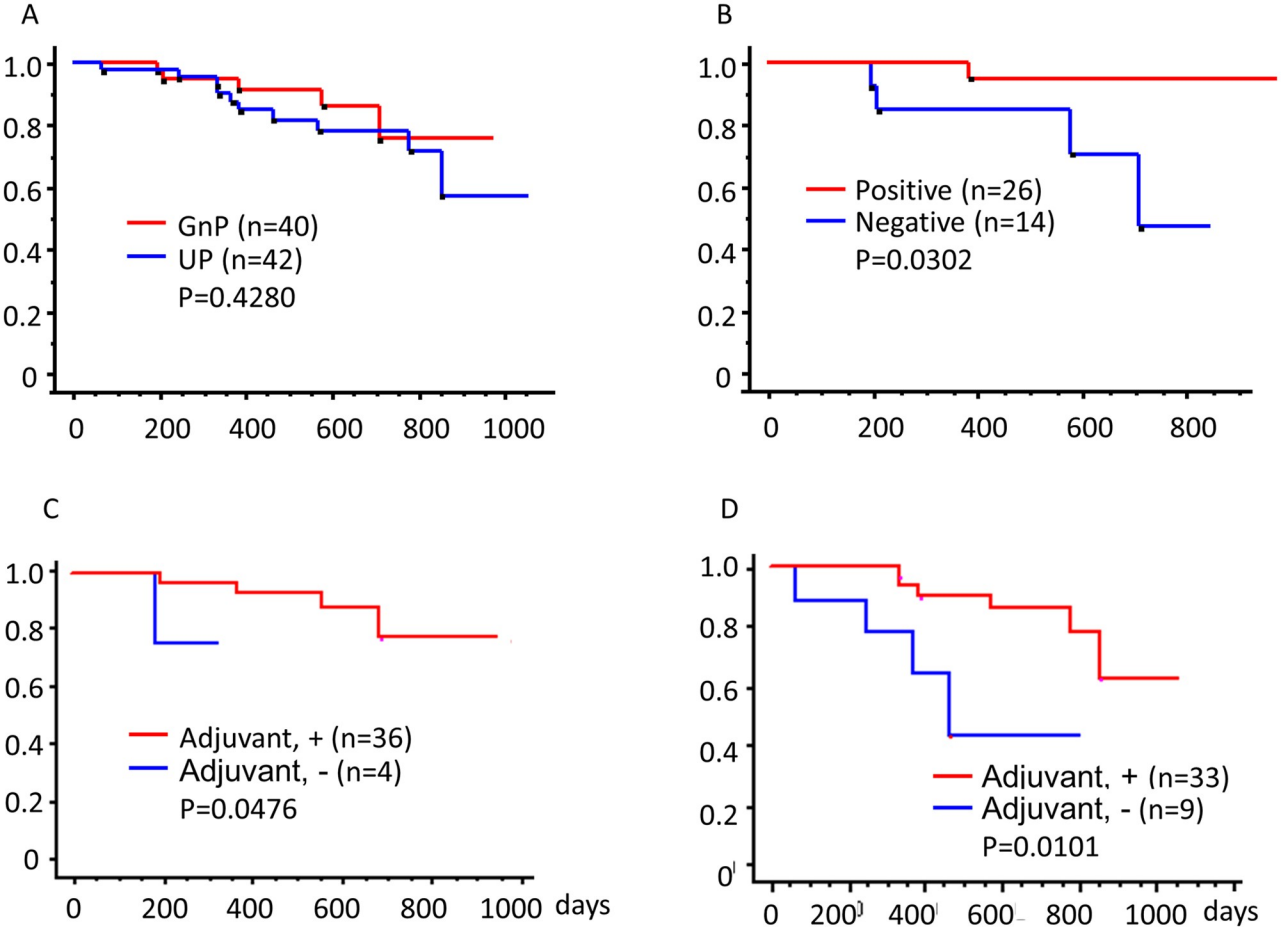
Overall survival of patients receiving neoadjuvant therapy. Kaplan-Meier survival analysis of 82 patients included in this study. (A) Red and blue curves represent patients receiving neoadjuvant therapy (gemcitabine combined with nab-paclitaxel, GnP group; n = 40) and those who underwent upfront surgery (UP group; n = 42), respectively. P = 0.4280. (B) Red and blue curves represent patients with encapsulating fibrosis (n = 26) and those without it (n = 14), respectively. P = 0.0302. (C) Red and blue curves represent patients receiving adjuvant therapy (S1, GnP, or Gem; n = 36) and patients without adjuvant therapy (n = 4), respectively in the GnP group. P = 0.0476. (D) Red and blue curves represent patients receiving adjuvant therapy (S1, GnP, or Gem; n = 33) and patients without adjuvant therapy (n = 9), respectively in the UP group. P = 0.0101.

Fig 4A shows Kaplan-Meier analysis of overall survival for GnP and UP patient groups. Although GnP group included patients with more advanced cancers at the time of diagnosis (Table 1), there was no statistically significant difference between the two study groups (P = 0.4280). Table 3 shows the results of univariate analysis based on Kaplan-Meier analysis of clinicopathological characteristics. Most patients showed a marked reduction in the levels of serum CA19-9 and tumor volume (median value of reduction rate, −92 and −46%, respectively). However, all clinical characteristics (including the rate of reduction in CEA level, CA19-9 level, and tumor volume) did not show any correlation with overall survival.

**Table 3:** Relationship between clinicopathological characteristics and overall survival in GnP patients.

		Number of cases	1 year survival rate (%)	P value
Sex	Male	15	90.0	0.8847
	Female	25	95.2	
Resectability	UR	2	100	N.D.
	BR	38	93.3	
Age	≥67	21	87.5	N.D.
	<67	19	100	
Reduction rate of CEA level (%)	≥-6.3	19	87.5	0.4820
	<-6.3	20	100	
Reduction rate of CA19-9 level (%)	≥-92	19	92.3	0.2471
	<-92	20	94.4	
Reduction rate of tumor volume (CT, %)	≥-46	20	92.9	0.1279
	<-46	20	94.1	
Adjuvant	+	36	80.1	0.0476*
	-	4^a^	0	
Histologic differentiation	Well	11	100	0.7028
	Moderately/poorly	29	90.9	
Stromal pattern	Scirrhous	5	66.7	0.3066
	Intermediate/Medurally	35	96.4	
T	3	38	93.1	N.D.
	4	2	100	
N	0	15	90.0	0.6699
	1	25	95.2	
Surgical margin	0 (Negative)	34	92.0	0.9576
	1 (Positive)	6	100	
Histologic evaluation of treatment effect				
Japan Pancreas Society	1a	19	88.2	0.9268
	1b	17	100	
	2	4	100	
Evans	I	19	88.2	0.9268
	IIa	17	100	
	IIb	4	100	
CAP	2	2	100	N.D.
	3	38	93.3	
Microscopic tumor volume (mm^3^)	≥16950	18	94.1	0.1749
	<16950	22	92.9	
No. of lymph nodes with metastasis	≥2	17	93.3	0.6699
	<2	23	93.8	
Tumor-stroma ratio (%)	≥40	22	94.1	0.5599
	<40	18	92.9	
Inflammation	Lymphoplasmacytic	9	100	0.7517
	Neutrophilic	31	91.3	
Fibrosis in the tumor	Mature	34	92.3	N.D.
	Immature	6	100	
Encapsulating fibrosis	0 (Negative)	14	81.8	0.0302*
	1 (Positive)	26	100	

*, P<0.05 by Kaplan-Meier analysis. Patients were divided into two groups according to median value of each parameter. Histologic evaluation of treatment effect according to criteria of the Japanese Pancreas Society, Evans, and College of American Pathologists (CAP). GnP, gemcitabine plus nab-paclitaxel; BR, borderline resectable; UR, unresectable; N.D., not determined.

^a^, Two patients were received chemotherapy as a palliative therapy.

The presence of encapsulating fibrosis showed a statistically significant correlation with overall survival (P = 0.0302). Patients with encapsulating fibrosis had a better prognosis than those without it (Fig 4B, P = 0.0302). However, other pathological characteristics did not show any correlation with overall survival. Most GnP patients showed mild treatment effects; in these cases, scores were determined using the criteria of the JPS (1a and 1b), Evans (I and IIa), and CAP (3).

For those in both GnP and UP groups, adjuvant chemotherapy showed an improvement in overall survival (Fig 4C, improved survival for the GnP group, P = 0.0476; Fig 4D, improved survival for the UP group, P = 0.0101). When we analyzed the association between adjuvant chemotherapy and encapsulating fibrosis, the number of patients was too small to analyze statistically.

### Differences between GnP patients with or without encapsulating fibrosis

We compared the clinicopathological characteristics of encapsulating fibrosis among patients with or without the condition. Patients with encapsulating fibrosis appeared to have lymphoplasmacytic inflammation, while patients without encapsulating fibrosis demonstrated the presence of neutrophilic infiltration (P = 0.0879, Table 4). Well-differentiated cancers with the scirrhous stromal pattern were more frequently observed in patients with encapsulating fibrosis than those without it; however, the differences were not statistically significant (patients with encapsulating fibrosis vs. patients without encapsulating fibrosis; well-differentiated, 35% vs. 14%, P = 0.35; scirrhous, 15% vs. 7%, P = 0.86).

**Table 4:** Characteristics of patients with encapsulating fibrosis in the GnP group.

	Negative	Positive	P-value
	14 cases	26 cases	
	Median (min-max)	Median (min-max)	
Age (years)	66.0 (36–76)	66.5 (39–81)	0.9209
Reduction rate of CEA levels	0.1 (-0.6–0.5)	1.5 (-0.8–2.9)	0.2357
Reduction rate of CA19-9 levels	-0.9 (-1.0–0.7)	-0.9 (-1.0–0.4)	0.9066
Reduction rate of tumor volume (CT)	-0.4 (-0.7–0.4)	-0.5 (-0.9–0)	0.1688
Microscopic tumor volume (mm^3^)	20675 (3250–46200)	16620 (825–209300)	0.6298
No. of lymph nodes with metastasis	1 (0–6)	1.3 (0–5)	0.7238
Necrosis (%)	10 (5–60)	5 (5–70)	0.4865
Tumor-stroma ratio (%)	50 (10–80)	35 (5–90)	0.1232
	Number (%)	Number (%)	
Sex			
Male	7 (50)	8 (31)	0.2308
Female	7 (50)	18 (69)	
Resectability			
BR	13 (93)	25 (96)	0.6482
UR	1 (7)	1 (4)	
Surgical margin			
0	13 (93)	21 (81)	0.3072
1	1 (7)	5 (19)	
T			
3	13 (93)	25 (96)	0.2080
4	1 (7)	1 (4)	
N			
0	5 (36)	10 (38)	0.8641
1	9 (64)	16 (62)	
Histologic differentiation			
Well	2 (14)	9 (35)	0.3496
Moderately	10 (71)	13 (50)	
Poorly	2 (14)	4 (15)	
Stromal pattern			
Intermediate	12 (86)	19 (73)	0.8580
Medullary	1 (7)	3 (12)	
Scirrhous	1 (7)	4 (15)	
Lymphatic invasion			
0	1 (7)	4 (15)	0.6398
1	11 (79)	20 (77)	
2	2 (14)	2 (8)	
Vascular invasion			
0	0 (0)	1 (4)	0.8456
1	9 (64)	18 (69)	
2	3 (21)	4 (15)	
3	2 (14)	3 (12)	
Histologic evaluation for treatment effect			
Japan Pancreas Society			
1a	6 (43)	13 (50)	0.7811
1b	6 (43)	11 (42)	
2	2 (14)	2 (8)	
Evans			
I	6 (43)	13 (50)	0.7811
IIa	6 (43)	11 (42)	
IIb	2 (14)	2 (8)	
CAP			
2	0 (0)	2 (8)	0.2870
3	14 (100)	24 (92)	
Inflammation			
Lymphoplasmacytic	1 (7)	8 (31)	0.0879
Neutrophilic	13 (93)	18 (69)	
Fibrosis in the tumor			
Mature	13 (93)	21 (81)	0.3072
Immature	1 (7)	5 (19)	

Scores were determined according to criteria of the Japan Pancreas Society, Evans, and College of American Pathologists (CAP). GnP, gemcitabine plus nab-paclitaxel; No., number CEA, carcinoembryonic antigen; CA19-9, carbohydrate antigen 19–9; CT, computed tomography.

## Discussion

**Fig 5:**
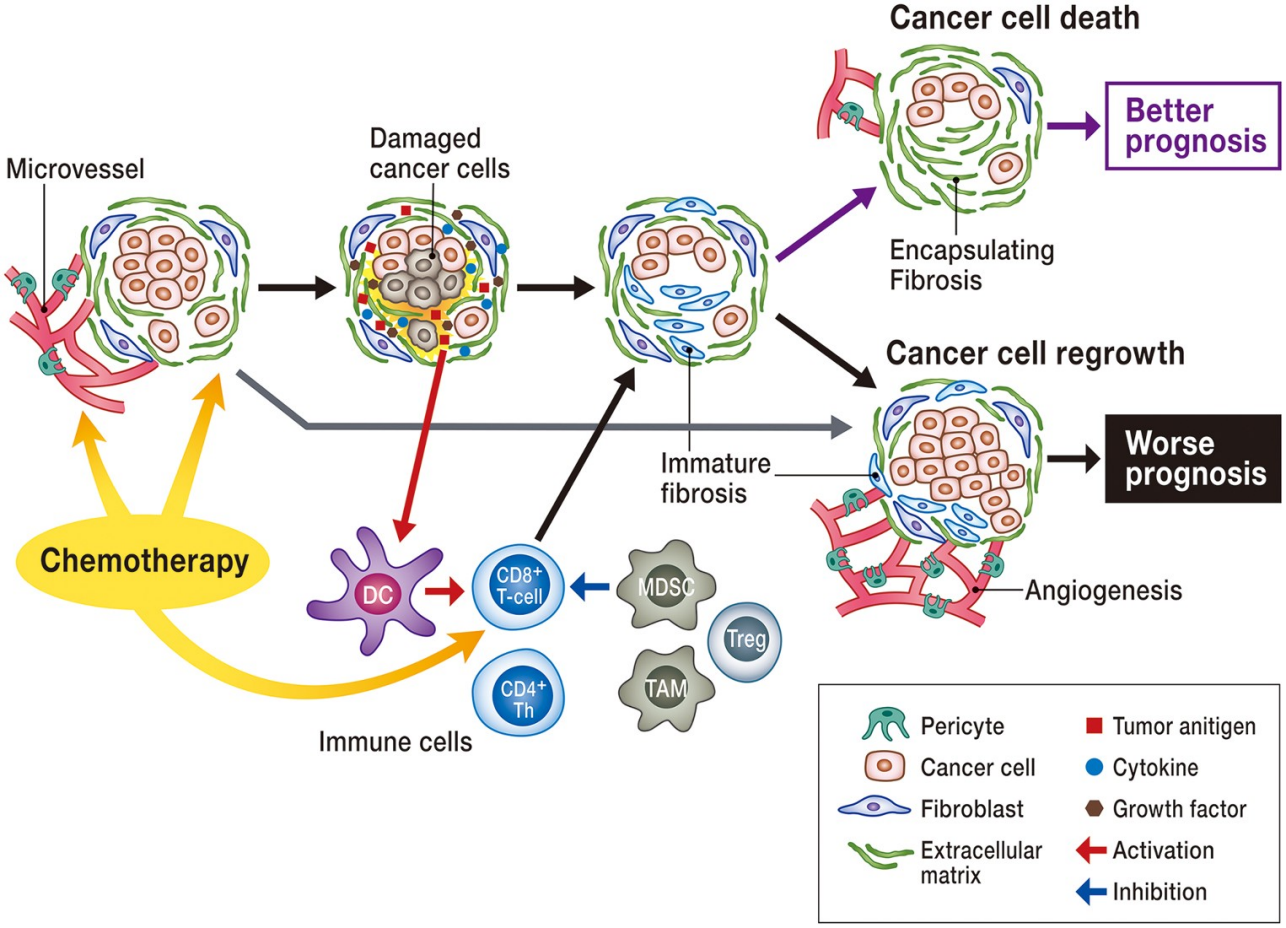
Schema of treatment-specific histologic changes in pancreatic cancer. Cancer tissue is composed of cancer cells and stromal fibroblasts. Chemotherapy causes damage to cancer cells, subsequently reducing the number of cancer cells and activated fibroblasts (low tumor-stroma ratio). As a result of cell death and long-lasting effects of chemotherapy, the number of activated fibroblasts decline, resulting in encapsulated mature fibrosis. When chemotherapy fails to kill cancer cells, cancer-associated fibroblasts continue to be activated, and this leads to poor prognosis. Other than fibroblasts, various cells (CD4, CD8 lymphocytes, macrophages, dendritic cells, endothelial cells, pericytes, etc.) and their crosstalk influence remnant cancer cells and sensitivity for chemotherapy.

In this study, we found that GnP induces a reduction in CA19-9 level, tumor volume, vascular invasion, lymph node metastasis, and tumor-stroma ratio, and high incidence of encapsulating mature fibrosis. Among these characteristics, encapsulating fibrosis appeared to be a good predictor of survival outcomes in patients with pancreatic cancer. In Fig 5, we have summarized our hypothesis regarding the development of fibrosis following NAT. Our results indicate that GnP can damage cancer cells, leading to decreased tumor-stroma ratio. A decrease in the number of cancer cells might induce inhibition of activated myofibroblasts, leading to mature fibrosis. Due to effectiveness of chemotherapy, such changes are expected to be more evident in the peripheral tumor areas, and this might result in the development of encapsulating fibrosis. Following NAT, when the cancer cells start to divide again or NAT fails to kill cancer cells, cancer-associated fibroblasts actively work to maintain the cancer microenvironment, leading to immature fibrosis. This might explain the poor prognosis. We believe that encapsulating fibrosis forms a tumor bed[26] at the same location that was populated by cancer cells before NAT. The presence of mature fibrosis in the cancer-surrounding area implies that NAT could possibly maintain cytotoxic effects for a long time, resulting in improved prognosis. Consistent with previous reports,[18] pathological changes associated with fibrosis are useful in predicting patient prognosis as they can facilitate accurate evaluation of treatment effects excluding tumor viability or necrotic changes.

Other than fibroblasts, various cells (CD4, CD8 lymphocytes, macrophages, dendritic cells, endothelial cells, pericytes, etc.) and their crosstalk influence remnant cancer cells and sensitivity for chemotherapy (Fig 5). Gemcitabine kills cancer cells and the endothelium [27], and suppresses cancer cell VEGF-A production [28]; thus, it can cause damage to microvessels in cancer tissue. Tumor microvessels have great influence on sensitivity of treatment. We found abundant microvessels in immature fibrosis while there were few microvessels in mature fibrosis.

Previous pathological studies have focused on the proportion of necrotic cancer cells resulting from NAT[29], where most of the patients received chemoradiotherapy[10, 14, 15, 17] and showed distinct histologic changes, such as necrosis. To our knowledge, this is the first report to show the prognostic value of pathologic evaluation of GnP treatment. According to previous tumor regression grading criteria, GnP treatment showed only mild effects; therefore, we distinguished the histologic changes induced by GnP treatment from tumor progression-related changes, for example, necrosis, inflammation, and fibrosis. We found that detection of necrosis was not useful in evaluating GnP treatment-specific pathological changes because the necrosis and inflammation were similar in GnP and UP patients. In contrast, the fibrosis patterns proved to be very helpful in studying the treatment effect of GnP.

Fibrosis is a fibroblast-dependent process that involves the formation of excess fibrous connective tissue and deposition of an extracellular matrix. Fibrosis can be a reactive, benign healing process; however, in cancerous tissues, fibrous stroma can form part of the cancer microenvironment, providing favorable conditions for tumor progression.[25] Fibroblasts can produce various growth factors and an extracellular matrix. In pancreatic cancer, cancer-associated fibroblasts support cancer cell progression and cause chemoresistance; therefore, fibroblast-targeting therapy is evolving as a new candidate for treating pancreatic cancer.[30] It is difficult to distinguish between NAT-related fibrosis and reactive fibrosis because pancreatic cancer is often associated with pancreatitis.[29] When we defined encapsulating fibrosis by the appearance of 2 mm band-like patterns of fibrosis, we could distinguish treatment effects from reactive changes. In addition, it is easy to evaluate by 10× magnification observation, because 2 mm is same as diameter of 10× magnification field. Miyashita et al. reported that GnP treatment lowers α-SMA-positive fibroblast density.[20] Furthermore, the same study showed that α-SMA density reflected a decrease in standardized uptake values of fluorodeoxyglucose on positron emission tomography (FDG-PET). Von Hoff et al. reported that GnP depleted the peritumoral desmoplastic stroma in patient-derived xenograft in mice.[31] Their data were compelling in athymic mice and they analyzed collagen type 1 fibers in stroma. In our study, we did not analyze neither α-SMA-positive myofibroblasts nor collagen type 1-positive stroma; however, we did examine the H&E-stained specimens to detect immature fibrosis (abundant fibroblasts) and mature fibrosis (few fibroblasts). There is a possibility that post-NAT, encapsulating fibrosis displays molecular characteristics that are different from non-treated cancer tissues. Thus, further research is needed to analyze the molecular characteristics of cancer-associated fibroblasts. Additionally, we found that there was a strong correlation between the tumor volumes determined by CT and microscopy. However, these techniques failed to predict patient outcome, suggesting that tumor diameter or volume cannot accurately predict the presence of cancer remnants because NAT alters the tumor-stroma ratio. Radiographic indicators, including Response Evaluation Criteria in Solid Tumors (RECIST) response, might not predict patient outcomes and treatment effects [30]; therefore, we need new point of view which is different from volumetric change using CT scan to estimate the stromal changes. We suppose virtual tactile evaluation such as shear wave elastography [32] using ultrasonography, or MR elastography could be candidates for assessment of peritumoral fibrosis.

Some pathological changes have been reported to result from NAT in pancreatic cancer patients, e.g., acellular mucous pools, macrophage aggregation, calcification, and a clear-cell variant of cancer.[14] We found that these findings are uncommon in GnP patients; therefore, they are not useful as prognostic markers. Inflammatory cells secrete various cytokines and play important roles in cancer cell growth and the development of fibrosis. It has been reported that nab-paclitaxel and gemcitabine induced a decrease in immunosuppressive myeloid-derived suppressor and regulatory T cell production [33]. In the present study, inflammatory infiltration did not show a statistically significant association with prognosis or fibrosis mainly due to small number of patients. Pancreatic cancer often causes pancreatitis due to rupture of pancreatic ducts, making it difficult to evaluate cancer-associated inflammatory cell infiltration. Further evaluation about the different immune cell response might be needed.

The present study has several limitations worth noting. Although valuable statistical comparisons were made, this was a retrospective study from a single institution involving two heterogeneous groups (GnP and UP) consisting of a low number of individuals that exhibited different clinical characteristics. There was a selection bias that resulted in the GnP group mainly consisting of patients with borderline resectable cancers while the UP group mainly comprised patients with resectable cancer. In addition, the overall survival analysis might be limited due to a short follow-up period (the median overall survival has not yet been reached, except for those (n = 14) without encapsulating fibrosis in the neoadjuvant group (n = 40)). However, our study has clarified pathological characteristics of GnP treatment, and provided useful contributions to establish pathological evaluation of effects of NAT in pancreatic cancer. We believe that encapsulating fibrosis might have strong power to predict patients’ outcomes. Thus, we intend to follow the present cohort in future and continue to collect more data to provide evidence for pathological evaluation of NAT effects. There are many treatment regimens for NAT, and our findings suggest that pathological changes might vary with different NAT regimens. Therefore, large cohort studies are warranted to further evaluate the prognostic value of encapsulating fibrosis, possibly with the help of imaging and biomarkers.
